# Emergency department visits among patients with neuromuscular diseases receiving home mechanical ventilation: a nationwide population-based study

**DOI:** 10.12701/jyms.2026.43.32

**Published:** 2026-05-12

**Authors:** Jae Hwa Bae, Soyoung Kwak

**Affiliations:** Department of Physical Medicine and Rehabilitation, Yeungnam University College of Medicine, Daegu, Korea

**Keywords:** Chronic respiratory failure, Emergency service, Mechanical ventilation, Pulmonary rehabilitation

## Abstract

**Background:**

Population-level data on emergency department (ED) utilization in patients with neuromuscular diseases (NMDs) receiving home mechanical ventilation (HMV) remain limited. This study investigated the frequency, outcomes, and associated factors of ED visits using a nationwide administrative database.

**Methods:**

Using the Health Insurance Review and Assessment Service database, we identified patients with NMDs who received HMV between January 2016 and October 2021, based on diagnostic codes and the MM441 procedure code. ED visits and outcomes were analyzed using claims data. Associations between ED visits and tracheostomy or gastrostomy were evaluated using the chi-square test.

**Results:**

A total of 1,569 patients were identified; amyotrophic lateral sclerosis (39.7%) and muscular dystrophy (31.0%) were the most common diagnoses. During the study period, 1,009 patients (64.3%) visited the ED at least once, accounting for 5,159 visits (mean 5.1 visits per patient). Among these visits, 35.9% resulted in hospitalization and 3.4% resulted in death in the ED. ED visits were more frequent among patients with tracheostomy than in those without tracheostomy (76.6% vs. 59.0%; relative risk [RR], 1.30; 95% confidence interval [CI], 1.21–1.39; *p*<0.001). In contrast, patients with gastrostomy had a lower proportion of ED visits than those without gastrostomy (22.6% vs. 55.1%; RR, 0.41; 95% CI, 0.35–0.48; *p*<0.001).

**Conclusion:**

ED utilization is common among patients with NMDs receiving HMV and is frequently associated with hospitalization, highlighting a substantial acute healthcare burden. These findings highlight the need for improved long-term respiratory care and outpatient management.

## Introduction

Recent advances in home mechanical ventilation (HMV) have substantially increased the number of patients receiving it worldwide. HMV has been shown to be effective in treating hypercapnic respiratory failure resulting from many conditions, including neuromuscular diseases (NMDs), chronic obstructive pulmonary disease, obesity hypoventilation syndrome, and restrictive lung disease [1,2].

Among patients receiving HMV, those with NMDs exhibit distinct clinical characteristics. NMD is a comprehensive term encompassing a variety of disorders affecting nerves, neuromuscular junctions, or muscles [3]. Despite their diverse etiologies, these disorders share common characteristics, including progressive muscle weakness and subsequent functional impairment. Progressive respiratory muscle weakness may lead to chronic respiratory failure and frequent aspiration due to secretion retention or difficulty swallowing. In addition, respiratory muscle weakness occurs in the context of generalized muscle involvement. Consequently, many patients with NMDs experience significant functional impairment and reduced mobility when ventilatory support becomes necessary, which may limit their ability to access routine outpatient care. Therefore, these patients are more likely to rely on emergency medical services when acute health issues arise [4].

Emergency department (ED) visits are relatively common among patients with chronic respiratory failure or ventilator dependence. Previous studies have shown that patients receiving home ventilatory support often present to the ED with respiratory complications, infections, device-related problems, or disease progression [4-8]. However, population-level data describing emergency healthcare utilization among patients with NMDs receiving HMV are limited. Most studies have been conducted in single centers or have focused on specific diseases such as amyotrophic lateral sclerosis (ALS), which may limit the generalizability of the findings and make it difficult to depict a broader landscape of unmet healthcare needs.

Therefore, we aimed to investigate the frequency, outcomes, and associated factors of ED visits among patients with NMDs receiving HMV using a nationwide administrative database.

## Methods

**Ethics statement:** The study protocol was approved by the Health Insurance Review and Assessment Service (HIRA) Public Data Provision Deliberation Committee before the data provision (M20220622005). This study was conducted according to the guidelines of the Declaration of Helsinki.

### 1. Data source

The HIRA database was used in this study. South Korea has a single-payer system, in which National Health Insurance covers the entire Korean population, and HIRA is responsible for reviewing medical claims, assessing healthcare quality and costs, and reimbursing healthcare providers. Therefore, the HIRA database contains all claims records for the entire population of South Korea, including sociodemographic information, diagnoses, health services provided, and provider information. Data from January 2016 to October 2021 were used in this study.

### 2. Patient selection and measures

Customized claims data from the HIRA database were used. HMV users with NMDs were identified using Korean Standard Classification of Diseases diagnosis codes for NMDs (i.e., E740, G120, G121, G1220, G1221, G1222, G1223, G1224, G1225, G1227, G1228, G129, G710, G711, G712, G713, G718, G719, G728, G729, G73, G731, G732, G733, G734, G735, G736, G737, and J986) (Table 1) and the MM441 procedure code (pulmonary rehabilitation for respiratory muscle dysfunction). MM441 is a procedure code for the evaluation of ventilatory status, adjustment of HMV, and education on HMV use in individuals with chronic ventilatory failure due to respiratory muscle dysfunction. Because the HIRA database does not contain specific procedures or diagnostic codes for HMV, patients with NMDs using HMV were identified by combining the MM441 procedure code with the disease codes listed in Table 1. Patients of all ages were included in the analysis. The study population included both incident and prevalent HMV users identified during the study period. Geographic distribution was defined based on the location where the MM441 procedure was performed, rather than the patients’ place of residence, to illustrate the distribution of healthcare service delivery, rather than the geographic distribution of the study population.

Data on ED visits were extracted using the claims data of the study population, which included patient demographic characteristics, principal and secondary diagnoses of the ED visits, and outcomes of the ED visits (i.e., discharge destinations, namely hospitalization, transfer to another hospital, patient death, and discharge to home). To identify the main reasons for ED visits, diagnostic codes corresponding to underlying NMDs were excluded because these codes were frequently recorded as the primary or secondary diagnosis regardless of the actual reason for the visit. The remaining diagnosis codes were categorized and analyzed according to frequency. Data from ED visits prior to HMV were excluded from the analysis.

Patients with tracheostomies were identified using the procedure codes O1301, O1302, and O1305 (invasive tracheostomy, percutaneous dilatational tracheostomy, and mediastinal tracheostomy, respectively), and patients with gastrostomies were identified using the procedure codes Q4911, Q4921, and Q4901 (percutaneous endoscopic gastrostomy, percutaneous radiologic gastrostomy, and open gastrostomy, respectively). Because these procedures are typically performed as long-term supportive interventions in patients with NMDs, they were considered proxies for the presence of tracheostomy or gastrostomy.

### 3. Statistical analysis

Patient characteristics are summarized using descriptive statistics and presented as frequencies and percentages. Associations between ED visits and tracheostomy and gastrostomy were analyzed using the chi-square test. All statistical analyses were performed using IBM SPSS ver. 25.0 (IBM Corp., Armonk, NY, USA) and *p*<0.05 was considered statistically significant.

## Results

### 1. Study population (primary diagnosis for home mechanical ventilation)

A total of 1,569 individuals with NMDs who received HMV during the study period were identified. ALS (623 patients, 39.7%) and muscular dystrophy (486 patients, 31.0%) were the most common primary diagnoses (Table 2). Of the 1,569 individuals, 1,045 (66.6%) were male and 524 (33.4%) were female (Table 3). Regarding age distribution, the largest proportion of individuals was observed in the 60 to 69-year age group (21.0%), followed by those aged 50 to 59 years (17.1%) and 20 to 29 years (17.0%). Individuals aged 70 to 79 years, 40 to 49 years, and 30 to 39 years represented 12.2%, 10.1%, and 9.5% of the study population, respectively (Table 3). Pediatric and adolescent patients comprised a smaller proportion of the sample, with 2.7% aged ≤9 years and 8.9% aged 10 to 19 years, respectively. Participants aged ≥80 years accounted for 1.5% of the total population.

Pulmonary rehabilitation for respiratory muscle dysfunction was predominantly performed in Seoul (62.8%), followed by Incheon (9.7%) and Daegu (7.3%) (Table 4). Other regions accounted for relatively small proportions, including Gyeonggi (6.0%), Jeolla (4.1%), Busan (3.5%), and Gyeongsang (2.9%). The remaining areas each comprised <2% of all procedures. Overall, pulmonary rehabilitation services were highly concentrated in the Seoul metropolitan area.

### 2. Emergency department visits in the study population

Of the 1,569 participants, 1,009 (64.3%) visited the ED at least once during the study period, accounting for a total of 5,159 visits (mean, 5.1 visits per patient; range, 1–51 visits per patient) (Table 5). Of the 1,009 participants, 667 (66.1%) were male, accounting for 69.7% of the 5,159 visits (Table 6). The highest proportion of visits occurred among individuals aged 60 to 69 years (24.5%) followed by those aged 50 to 59 years (20.5%). Participants aged ≥80 years represented the smallest proportion of both participants and visits (Table 6). The outcomes of the ED visits are summarized in Table 7. Among the 5,159 visits, discharge to home was the most common outcome (52.6%), followed by hospitalization (35.9%), transfer to another facility (8.2%), and death in the ED (3.4%) (Table 7).

ED visits were further analyzed according to underlying NMDs (Table 8). Among the 1,009 participants who visited the ED at least once, ALS was the most common diagnosis, accounting for 422 individuals (41.8%) and 2,317 ED visits (44.9%). Muscular dystrophy accounted for 276 participants (27.4%) and 1,345 visits (26.1%), followed by other motor neuron diseases (112 participants [11.1%] and 628 visits [12.2%]). Spinal muscular atrophy accounted for 53 participants (5.3%) and 237 visits (4.6%), while other diagnoses accounted for smaller proportions. After excluding the underlying NMD codes, pneumonia and chronic respiratory failure were the most frequent principal diagnoses recorded during the ED visits. For the secondary diagnoses recorded during ED visits, tracheostomy, gastrostomy, and dysphagia were ranked, excluding pneumonia and chronic respiratory failure.

Among the 1,569 participants included in the study, 478 (30.5%) underwent tracheostomy and 650 (41.4%) underwent gastrostomy. ED visits were more frequent among patients with tracheostomy than among those without tracheostomy (76.6% vs. 59.0%; relative risk [RR], 1.30; 95% confidence interval [CI], 1.21–1.39; *p*<0.001). In contrast, patients with gastrostomy had a lower proportion of ED visits when compared to those without gastrostomy (22.6% vs. 55.1%; RR, 0.41; 95% CI, 0.35–0.48; *p*<0.001) (Table 9).

## Discussion

This nationwide population-based study identified 1,569 patients with NMDs who received HMV. A recent study using the National Health Interview Survey database indicated that 4,785 patients were prescribed HMV between August 2015 and July 2017, and approximately 42% of them had NMDs [9]. In that study, the category of NMD included a broad spectrum of conditions, such as Guillain–Barré syndrome, myasthenia gravis, and peripheral neuropathies. In contrast, the present study identified patients using a combination of NMD diagnostic codes and the MM441 procedure code, which is used to evaluate and manage chronic ventilatory failure due to respiratory muscle dysfunction. Because the MM441 code is typically applied in the context of ongoing ventilatory management and follow-up, patients with acute or potentially reversible neuromuscular conditions who recovered their respiratory function and were successfully weaned from mechanical ventilation within a relatively short period may have been less likely to be captured in our dataset. Consequently, the present cohort might represent a population with chronic and persistent ventilatory support requirements. Male patients accounted for approximately two-thirds of the study population, with the largest age group comprising individuals in their 60s. This distribution likely reflects the epidemiological characteristics of the major underlying NMDs in this cohort. ALS, the most common diagnosis in our study population, typically occurs in middle-aged and older adults and has a slight male predominance [10]. In addition, muscular dystrophies, such as Duchenne and Becker muscular dystrophies, show male predominance. Advances in multidisciplinary care, including the widespread use of corticosteroids and cardioprotective agents, as well as ventilatory support using HMV, have improved survival and delayed the onset of severe respiratory failure in patients with Duchenne muscular dystrophy. Consequently, the peak age of severe respiratory impairment requiring ventilatory support has shifted from adolescence to early adulthood [11,12].

The study also showed that more than 60% of the MM441 procedures were performed in Seoul, whereas the city accounted for only approximately 20% of the total population of South Korea [13]. Given that patients with advanced NMDs often have mobility limitations, such geographic concentration may create barriers to access for patients living in other regions. These findings suggest the need to strengthen the regional healthcare capacity and improve access to specialized respiratory care services for patients with HMV. However, because data on the patients’ residential locations were not available, the extent to which this distribution reflects disparities in patient access remains uncertain.

This study showed that ED visits were common among patients with NMDs receiving HMV. Nearly two-thirds of the patients (1,009 of 1,569) visited the ED at least once during the study period. In addition, more than one-third of ED visits led to hospitalizations, which is a higher rate considering that only 10% to 20% of patients visiting the ED in the general population are hospitalized [14,15]. This finding is consistent with that of a recent nationwide study examining ED utilization among patients with ALS in Korea [4].

In our analysis, the presence of tracheostomy was associated with ED visits, because the need for a tracheostomy might reflect more advanced disease. Tracheostomy may also be associated with a higher risk of complications related to airway secretion, tube obstruction, infection, and tube dislodgment [16]. Conversely, patients with gastrostomy were less likely to visit the ED. One possible explanation is that enteral feeding via gastrostomy may improve nutritional status and reduce the risk of aspiration associated with oral feeding [17-19]. However, this finding should be interpreted with caution because gastrostomy placement may indicate that these patients had better access to multidisciplinary care or that their caregivers were better educated in patient management, rather than a direct protective effect of gastrostomy.

This study had several limitations. First, because it was conducted based on administrative claims data, detailed clinical information, such as disease severity, ventilator settings, functional status, and exact reasons for the emergency room visits, was not available. Therefore, it was not possible to perform a multivariable analysis to adjust for potential confounding factors. For similar reasons, it was not possible to obtain detailed data at the individual level, preventing us from conducting a more in-depth analysis of the characteristics of patients who frequently visited the ED. Second, owing to the limitations of this observational study, the correlation between tracheostomies or gastrostomies and ED visits could not be clearly determined. Third, although the HIRA database provides data covering the entire population of South Korea, it is not free of coding errors; therefore, diagnostic or procedure codes may have been entered incorrectly. Future studies incorporating more detailed clinical variables and longitudinal data may help further elucidate the factors associated with ED utilization in this population.

Despite these limitations, to the best of our knowledge, this is the first study to provide important information regarding ED visits among patients with NMDs receiving HMV. This study may help inform the development of tailored treatment strategies for this population and guide the design of home-based medical care service programs for homebound patients, including physician home visits and telemonitoring.

## Figures and Tables

**Table 1.** Diseases used to identify neuromuscular disease in the study (Korean Standard Classification of Diseases, 7th revision)

Code	Disease
E740	Glycogen storage disease
G120	Infantile spinal muscular atrophy, type I
G121	Other inherited spinal muscular atrophy
G1220	Familial amyotrophic lateral sclerosis
G1221	Sporadic amyotrophic lateral sclerosis
G1222	Primary lateral sclerosis
G1223	Progressive bulbar palsy
G1224	Progressive muscular atrophy
G1225	X-linked spinobulbar muscular atrophy
G1227	Other genetic or hereditary motor neuron disease
G1228	Other and unspecified motor neuron disease
G129	Spinal muscular atrophy, unspecified
G710	Muscular dystrophy
G711	Myotonic disorders
G712	Congenital myopathies
G713	Mitochondrial myopathy, NEC
G718	Other primary disorders of muscles
G719	Primary disorder of muscle, unspecified
G728	Other specified myopathies
G729	Myopathy, unspecified
G73	Disorders of myoneural junction and muscle in diseases classified elsewhere
G731	Lambert-Eaton syndrome
G732	Other myasthenic syndromes in neoplastic disease
G733	Myasthenic syndromes in other diseases classified elsewhere
G734	Myopathy in infectious and parasitic disease classified elsewhere
G735	Myopathy in endocrine diseases
G736	Myopathy in metabolic diseases
G737	Myopathy in other diseases classified elsewhere
J986	Disorders of diaphragm

**Table 2.** Primary diagnoses for pulmonary rehabilitation for respiratory muscle dysfunction

Disease	Data
Motor neuron disease	865 (55.1)
Amyotrophic lateral sclerosis	623 (39.7)
Spinal muscular atrophy	84 (5.4)
Progressive bulbar palsy	8 (0.5)
Other motor neuron disease	150 (9.6)
Muscular dystrophy	486 (31.0)
Myotonic disorders	113 (7.2)
Myopathy	83 (5.3)
Congenital myopathy	35 (2.2)
Mitochondrial myopathy	24 (1.5)
Other and unspecified primary disorder of muscle	24 (1.5)
Glycogen storage disease	11 (0.7)
Disorders of diaphragm	11 (0.7)

Values are presented as number (%).

**Table 3.** Basic characteristics of individuals who received pulmonary rehabilitation for respiratory muscle dysfunction (MM441)

Characteristic	Data
Sex	
Male	1,045 (66.6)
Female	524 (33.4)
Age group (yr)	
0–9	42 (2.7)
10–19	139 (8.9)
20–29	267 (17.0)
30–39	149 (9.5)
40–49	158 (10.1)
50–59	269 (17.1)
60–69	329 (21.0)
70–79	192 (12.2)
≥80	24 (1.5)

Values are presented as number (%).

**Table 4.** Geographic distribution of locations where pulmonary rehabilitation for respiratory muscle dysfunction was delivered

Area	Data
Seoul	985 (62.8)
Busan	55 (3.5)
Incheon	152 (9.7)
Daegu	115 (7.3)
Gwangju	6 (0.4)
Daejeon	23 (1.5)
Ulsan	13 (0.8)
Gyeonggi	94 (6.0)
Gangwon	3 (0.2)
Chungcheong	10 (0.6)
Jeolla	65 (4.1)
Gyeongsang	46 (2.9)
Jeju	2 (0.1)
Total	1,569 (100)

Values are presented as number (%).

**Table 5.** Emergency department visits during the study period

Variable	Data
Total number of participants	1,569
Participants with ≥1 emergency department visit	1,009 (64.3)
Total number of emergency department visits	5,159
Mean number of visits per participant with ≥1 visit	5.1
Range of visits per participant	1–51

Values are presented as number or number (%).

**Table 6.** Basic characteristics of individuals who visited the emergency department at least once during the study period

Variable	No. of participants	No. of visits
Sex		
Male	667 (66.1)	3,598 (69.7)
Female	342 (33.9)	1,561 (30.3)
Age group (yr)		
0–9	32 (3.2)	163 (3.2)
10–19	81 (8.0)	344 (6.7)
20–29	159 (15.8)	819 (15.9)
30–39	85 (8.4)	329 (6.4)
40–49	96 (9.5)	486 (9.4)
50–59	184 (18.2)	1,056 (20.5)
60–69	231 (22.9)	1,265 (24.5)
70–79	129 (12.8)	661 (12.8)
≥80	12 (1.2)	36 (0.7)

Values are presented as number (%).

**Table 7.** Outcome after emergency department visits

Disposition	No. of visits
Discharge home	2,713 (52.6)
Hospitalization	1,851 (35.9)
Transfer to another facility	421 (8.2)
Death in the emergency department	174 (3.4)
Total	5,159 (100)

Values are presented as number (%).

**Table 8.** Emergency department visits classified by primary diagnoses for pulmonary rehabilitation for respiratory muscle dysfunction

Disease	No. of participants receiving MM441	No. of participants receiving MM441 with ≥1 ED visits	No. of ED visits
Motor neuron disease	865 (55.1)		
Amyotrophic lateral sclerosis	623 (39.7)	422 (41.8)	2,317 (44.9)
Spinal muscular atrophy	84 (5.4)	53 (5.3)	237 (4.6)
Progressive bulbar palsy	8 (0.5)	6 (0.6)	26 (0.5)
Other motor neuron disease	150 (9.6)	112 (11.1)	628 (12.2)
Muscular dystrophy	486 (31.0)	276 (27.4)	1,345 (26.1)
Myotonic disorders	113 (7.2)	67 (6.6)	291 (5.6)
Myopathy	83 (5.3)		
Congenital myopathy	35 (2.2)	24 (2.4)	121 (2.4)
Mitochondrial myopathy	24 (1.5)	17 (1.7)	64 (1.2)
Other and unspecified primary disorder of muscle	24 (1.5)	17 (1.7)	44 (0.9)
Glycogen storage disease	11 (0.7)	8 (0.8)	26 (0.5)
Disorders of diaphragm	11 (0.7)	7 (0.7)	60 (1.2)
Total	1,569 (100)	1,009 (100)	5,159 (100)

Values are presented as number (%).

**Table 9.** Association between medical procedures and emergency department (ED) visits

Procedure	ED visit	No ED visit	Relative risk (95% CI)	*p*-value
Tracheostomy				
Present	366	112	1.30 (1.21–1.39)	<0.001
Absent	643	448		
Gastrostomy				
Present	147	503	0.41 (0.35–0.48)	<0.001
Absent	506	413		

CI, confidence interval.
